# Engineering of leucine-responsive regulatory protein improves spiramycin and bitespiramycin biosynthesis

**DOI:** 10.1186/s12934-019-1086-0

**Published:** 2019-02-19

**Authors:** Zhili Lu, Xiaoting Zhang, Jianlu Dai, Yiguang Wang, Weiqing He

**Affiliations:** 1 NHC Key Laboratory of Biotechnology of Antibiotics, Institute of Medicinal Biotechnology, Chinese Academy of Medical Sciences, No. 1 Tian Tan Xi Li, Beijing, 100050 People’s Republic of China; 2 State Key Laboratory of Respiratory Disease, Guangzhou Regenerative Medicine and Health Guangdong Laboratory, Guangzhou Institutes of Biomedicine and Health, Chinese Academy of Sciences, Guangzhou, 510530 China

**Keywords:** Spiramycin, Bitespiramycin, Leucine-responsive regulatory protein, Branched-chain amino acids

## Abstract

**Background:**

Bitespiramycin (BT) is produced by recombinant spiramycin (SP) producing strain *Streptomyces spiramyceticus* harboring a heterologous 4″-O-isovaleryltransferase gene (*ist*). Exogenous l-Leucine (l-Leu) could improve the production of BT. The *orf2* gene found from the genomic sequence of *S. spiramyceticus* encodes a leucine-responsive regulatory protein (Lrp) family regulator named as SSP_Lrp. The functions of SSP_Lrp and l-Leu involved in the biosynthesis of spiramycin (SP) and BT were investigated in *S. spiramyceticus*.

**Results:**

SSP_Lrp was a global regulator directly affecting the expression of three positive regulatory genes, *bsm23*, *bsm42* and *acyB2*, in SP or BT biosynthesis. Inactivation of SSP_Lrp gene in *S. spiramyceticus* 1941 caused minor increase of SP production. However, SP production of the Δ*SSP_Lrp*-SP strain containing an SSP_Lrp deficient of putative l-Leu binding domain was higher than that of *S. spiramyceticus* 1941 (476.2 ± 3.1 μg/L versus 313.3 ± 25.2 μg/L, respectively), especially SP III increased remarkably. The yield of BT in Δ*SSP_Lrp-*BT strain was more than twice than that in 1941-BT. The fact that intracellular concentrations of branched-chain amino acids (BCAAs) decreased markedly in the Δ*SSP_Lrp*-SP demonstrated increasing catabolism of BCAAs provided more precursors for SP biosynthesis. Comparative analysis of transcriptome profiles of the Δ*SSP_Lrp*-SP and *S. spiramyceticus* 1941 found 12 genes with obvious differences in expression, including 6 up-regulated genes and 6 down-regulated genes. The up-regulated genes are related to PKS gene for SP biosynthesis, isoprenoid biosynthesis, a Sigma24 family factor, the metabolism of aspartic acid, pyruvate and acyl-CoA; and the down-regulated genes are associated with ribosomal proteins, an AcrR family regulator, and biosynthesis of terpenoid, glutamate and glutamine.

**Conclusion:**

SSP_Lrp in *S. spiramyceticus* was a negative regulator involved in the SP and BT biosynthesis. The deletion of SSP_Lrp putative l-Leu binding domain was advantageous for production of BT and SP, especially their III components.

**Electronic supplementary material:**

The online version of this article (10.1186/s12934-019-1086-0) contains supplementary material, which is available to authorized users.

## Background

**Fig. 1:**
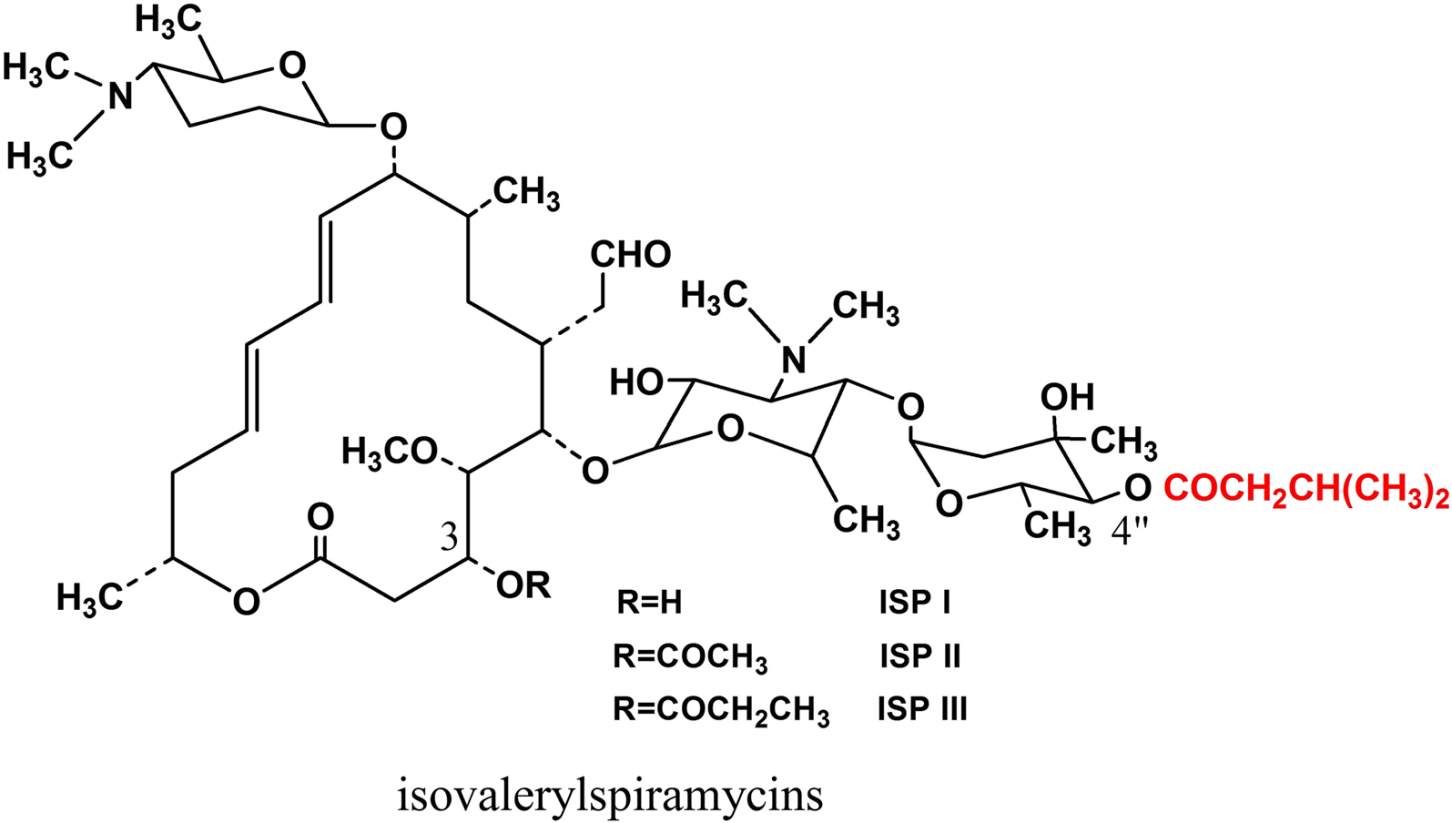
Chemical structure of isovalerylspiramycins

Bitespiramycin (biotechnological spiramycin, BT), new genetic engineering 16-membered macrolide antibiotic, is a group of 4″-acylated spiramycins with three 4″-isovalerylspiramycins (ISP; Fig. 1) as its major components, produced by recombinant *S. spiramyceticus* harboring a 4″-O-isovaleryltransferase gene (*ist*) from a carbomycin producer, *Streptomyces thermotolerans* [1]. ISP has three components due to low specificity of 3-O-acytransferase involved SP biosynthesis. SP is produced as a mixture of three major compounds differing by acyl and propionyl substitutions at the position of the hydroxyl group at carbon 3 [2]. Compared with SP, BT has a longer half-life time, higher potency, and better tissue penetration and pharmacokinetic characteristics [3, 4] and is currently being evaluated for approval by the State Food and Drug Administration of China. The quality standard of BT requires the isovalerylspiramycin I, II and III more than 80%, and more than 30% on isovalerylspiramycin III. The 4″-isovaleryl group of BT originates from the l-Leucine (l-Leu) metabolic pathway. So, branched-chain amino acids, particularly l-Leu, can significantly improve the yield of BT [5]. An effective way to develop high-producing BT strain that produces more l-Leu is to eliminate the feedback regulation caused by l-Leu itself as end product. The engineering of relevant regulatory genes to modulate transcriptional activation or inhibition is an effective way to improve the yield of target secondary metabolites in Streptomyces [6, 7].

Leucine-responsive regulatory proteins (Lrps) are a group of transcriptional regulators that modulate diverse cellular processes in bacteria and archaea. The C-terminal ligand binding domain of Lrp adopts a βαββαβ fold in which four-stranded antiparallel -sheets are flanked by two α-helices [8]. Lrps are widely distributed among prokaryotes and control diverse cellular processes [9–12]. Moreover, it has been reported that Lrp controlled the expression of many genes directly or indirectly involved in diverse metabolisms, such as branched-chain amino acid metabolism, pili synthesis, virulence repressor, polyamine homeostasis, and methanol assimilation [13–17]. The activity of Lrp on target genes is modulated by the binding of l-Leu to the C-terminal region of Lrp. l-Leu often acts as a signal for the availability of nutrients to cells, and can have both positive and negative effects upon the regulatory output of Lrp [18].

Lrp homologs from several organisms have been shown to be responsive to a variety of amino acids; for example, expression of the *alaE* gene is positively regulated by Lrp in response to intracellular accumulation of l-alanine in *Escherichia coli* [19]. Additionally, intracellular branched-chain amino acids (BCAAs) increase when the ligand-sensing region in the C-terminal domain of Lrp was deleted in *Glyptostrobus europaeus* [20]. However, there were only a few studies on the regulatory role of Lrp in antibiotic biosynthesis. The Lrp family regulator SACE_Lrp was found to regulate the transport and catabolism of BCAAs, thereby playing an important role in regulating erythromycin production in *Saccharopolyspora erythraea* [21]. Additionally, SCO3361, an Lrp/AsnC family regulator, was found to control actinorhodin production and morphological development in *Streptomyces coelicolor* [22].

Extensive genetic and biochemical studies have identified the genes involved in SP biosynthesis in *Streptomyces ambofaciens* [2, 23–25]. The SP gene cluster contains 41 genes arranged in four major polycistronic units, including three positive regulatory genes, *srm22*, *srm40* and *saaR*, in *S. ambofaciens* [23, 26]. The AcyB2 was a positive regulator for transcription of the *ist* gene derived from the carbomycin biosynthetic gene cluster in *S. thermotolerans* [27, 28]. Genomic DNA of *S. spiramyceticus* 1941 has been fully sequenced which assisted to find out the gene cluster of SP biosynthesis. The *lrp* gene is located about 4.5 Mb from the SP gene cluster in *S. spiramyceticus* 1941.

In this study, we aimed to investigate the functions of and mechanisms through which Lrp controls SP and BT production in *S. spiramyceticus* 1941. Our results provided novel insights into the vital role of Lrp in the biosynthesis of these significant antibiotics.

## Results

### The Lrp gene in *S. spiramyceticus* 1941

**Fig. 2:**
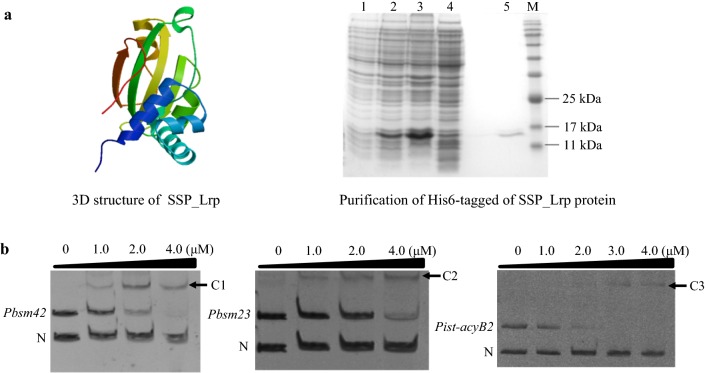
The predicted domains and three-dimensional (3D) structure of SSP_Lrp. **a** 3D structure and SDS-PAGE detection of His6-tagged SSP_Lrp protein. **b** EMSAs of DNA binding of Lrp to *Pbsm42*, *Pbsm23*, and *Pist-acyB2*. C1: *Pbsm42*-SSP_Lrp complex; C2: *Pbsm23*-SSP_Lrp complex; C3: *Pist-acyB2*-SSP_Lrp complex; N: nonspecific control DNA. *P < 0.05, **P < 0.01

There are three positive regulatory genes to be found in the BT biosynthetic gene cluster (GenBank accession number: MH460451) including *acyB2* (GenBank accession number: D31821.1 or KR818745), *bsm42* (homologous to *srm40* in *Streptomyces ambofaciens*), and *bsm23* (homologous to *srm22* in *S. ambofaciens*) (unpublished data). Their promoters sequence all contained the conserved motif of Lrp 5′-YAGHAWATTWTDCTR-3′ (Y = C or T, H = not G, W = A or T, D = not C, and R = A or G) [29]. By genomic alignment of *S. spiramyceticus* 1941, *orf2* (GenBank accession number: MH460452), a Lrp family homolog designating SSP_Lrp, exhibits 93% and 34% amino acid identity with a Lrp/AsnC family transcriptional regulators in *Streptomyces flavidovirens* and SACE_Lrp in *S. erythraea*, respectively. Three-dimensional structure SSP_Lrp of 148 amino acid residues was predicted by (PS)2v2 (Protein Structure Prediction Server) online software, as shown in Fig. 2a. The N-terminal domain of the helix-turn-helix is considered a DNA binding domain, and the C-terminal domain contains five β-sheets and two α-helix intervals, potentially acting in l-Leu cofactor binding in this domain.

### Expression of SSP_Lrp in *E. coli* BL21 (DE3) and electrophoretic mobility shift assay (EMSA)

His6-tagged SSP_Lrp was expressed in *E. coli* BL21 (DE3) (Fig. 2a), and its affinity for promoter sequences of regulatory genes involved in SP and BT biosynthesis was examined by EMSA. EMSAs were carried out between the purified SSP_Lrp protein and the *Pist-acyB2*, *Pbsm23*, and *Pbsm42* promoter fragments (Fig. 2b). The results showed that SSP_Lrp protein could specifically bind to the promoters of *bsm42*, *bsm23*, and *ist-acyB2* to form specific protein-DNA complexes. Thus, SSP_Lrp and promoter complexes formed in a concentration-dependent manner, indicating that SSP_Lrp could modulate these positive regulatory genes affecting SP and BT biosynthesis.

### Role of the SSP_Lrp in SP and BT biosynthesis

**Fig. 3:**
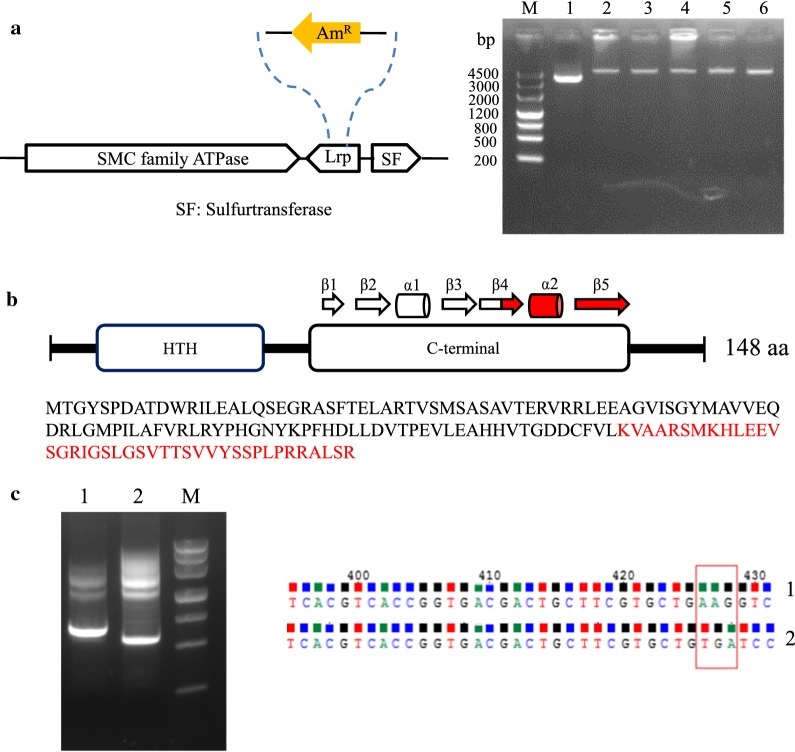
Identification of the Δ*SSP_Lrp* and Δ*SSP_Lrp*-SP mutant. **a** The SSP_Lrp gene inactivated by insertion of the aparamycin resistance cassette, 1: *S. spiramyceticus* 1941, 2–6: Δ*SSP_Lrp*; **b** design of the SSP_Lrp deletion (shown in red) in the C-terminal. **c** Confirmation of Δ*SSP_Lrp*-SP by PCR and sequencing. 1: *S. spiramyceticus* 1941; 2: Δ*SSP_Lrp*-SP mutant

Inactivation of the SSP_Lrp gene by apramycin (Am) resistance gene cassette insertion was confirmed by PCR by the *lrp*-DF/DR primers which were designed outside the homologous arm sequence. The PCR product in the *S. spiramyceticus* 1941 was smaller than that in the ∆*SSP_Lrp* mutant (Fig. 3a). The Δ*SSP_Lrp*-SP strain contains the truncated SSP_Lrp gene with deletion on the putative l-Leu binding sequence (120 bp), which was confirmed by PCR amplified by *lrp*-CF/R primer and DNA sequencing (Fig. 3b, c).

**Fig. 4:**
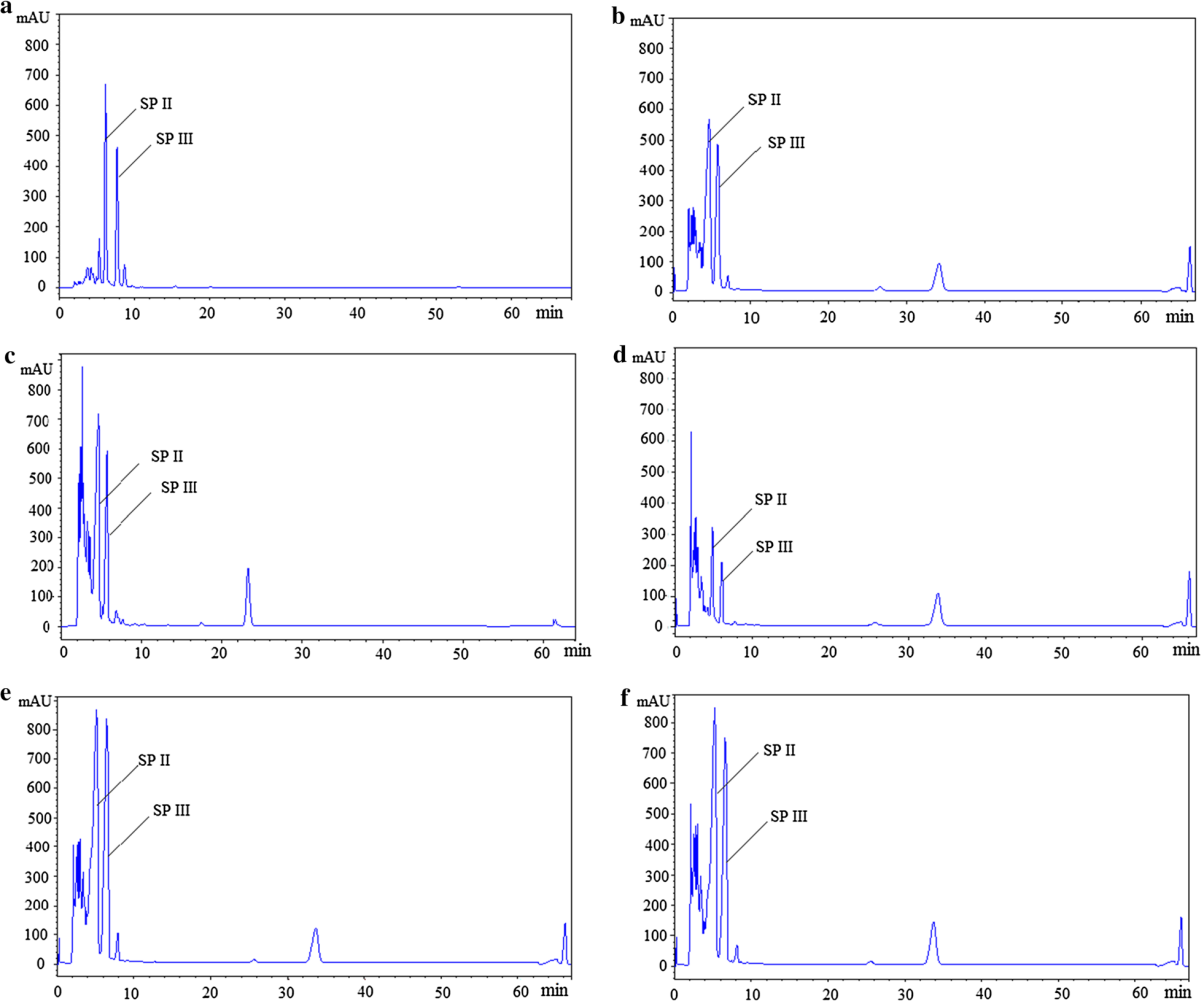
HPLC detection of SP in *S. spiramyceticus* 1941 and SSP_Lrp gene mutants. **a** spiramycin, **b** *S. spiramyceticus* 1941, **c** Δ*SSP_Lrp*, **d** 1941-C, **e** Δ*SSP_Lrp*-SP, F: Δ*SSP_Lrp*-SP–C

SP is the only antibacterial compound in present fermentation conditions for *S. spiramyceticus* 1941. In order to characterize the possible regulatory role of the SSP_Lrp gene in SP biosynthesis, antibacterial assays were performed in the following mutants: (1) *S. spiramyceticus* 1941; (2) 1941-C (SSP_Lrp overexpression in *S. spiramyceticus* 1941); (3) ∆*SSP_Lrp*-SP (truncated SSP_Lrp gene); (4) ∆*SSP_Lrp*-SP-C (SSP_Lrp overexpression in ∆*SSP_Lrp*-SP strain); (5) ∆*SSP_Lrp*. The antibacterial activities of fermentation products from *S. spiramyceticus* 1941 and its mutants were evaluated by measuring the *Bacillus subtilis* growth inhibition zone (Table 1). The inhibition zones of ∆*SSP_Lrp*, ∆ *SSP_Lrp*-SP and Δ*SSP_Lrp*-SP-C were larger than that of *S. spiramyceticus* 1941, and the ∆*SSP_Lrp*-SP mutant displayed the best antibacterial activity. Interestingly, however, antibacterial circles became much smaller in broth of 1941-C mutants. The SP production detected by HPLC in the wild-type strain and these mutants showed the similar pattern (Fig. 4). Compared with that in the wild-type stain, the SP concentration was significantly decreased in the 1941-C mutants. The production of SP, especially the SP III component, apparently increased in Δ*SSP_Lrp*-SP and Δ*SSP_Lrp*-SP-C mutants.

**Table 1:** The zone of inhibition tests on the fermentation broths of the wild-type and different SSP_Lrp mutants

Strains	Characteristics	Zone of inhibition (mm)
1941	*S. spiramyceticus* 1941 (wild type)	19.5 ± 1.1
1941-C	1941::ermEp*-*SSP_Lrp*	15.2 ± 0.6
Δ*SSP_Lrp*-SP	Truncated SSP_Lrp at the C terminal	21.6 ± 0.5
Δ*SSP_Lrp* -SP–C	Δ*SSP_Lrp*-SP::ermEp*-*SSP_Lrp*	20.1 ± 0.7
Δ*SSP_Lrp*	Inactivation of the SSP_Lrp gene	20.8 ± 0.8

**Fig. 5:**
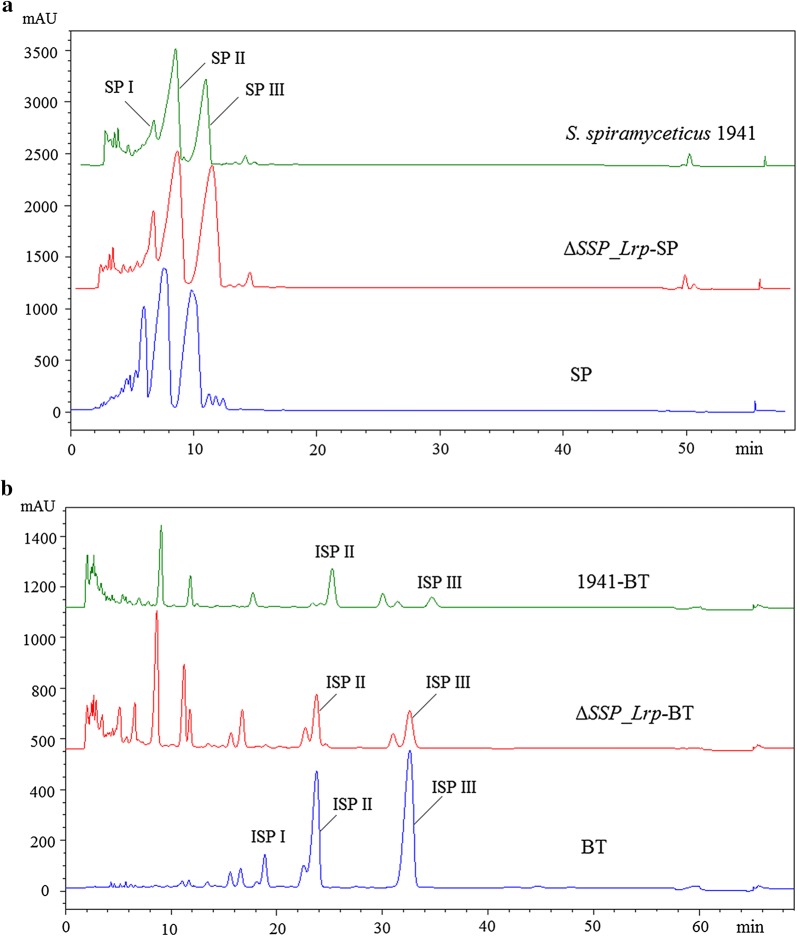
HPLC detection of SP and ISP production in the four strains. **a** Comparison of SP production between Δ*SSP_Lrp*-SP mutant and *S. spiramyceticus* 1941 strains; **b** comparison of ISP production between Δ*SSP_Lrp*-BT and 1941-BT strains

Next, we quantitatively compared the fermentation products of the ∆*SSP_Lrp*-SP mutant and wild-type strain by HPLC (Fig. 5a and Table 2). SP typically contained three components: SP I, SP II, and SP III. SP production of the ∆*SSP_Lrp*-SP mutant was obviously higher than that of *S. spiramyceticus* 1941 (476.2 ± 3.1 μg/L versus 313.3 ± 25.2 μg/L, respectively). Moreover, compared with the wild-type strain (24.4%), the proportion of SP III in the ∆*SSP_Lrp*-SP mutant increased to 33%.

**Table 2:** HPLC detection of SP and BT production in the four strains

Strain	SP I or ISP I (%)	SP II or ISP II (%)	SP III or ISP III (%)	SP or ISP (μg/L)
*S. spiramyceticus* 1941	12.0 ± 0.3	39.9 ± 1.8	24.4 ± 2.9	313.3 ± 25.2
Δ*SSP_Lrp*-SP	11.1 ± 1.8	37.4 ± 0.8	33.0 ± 0.1	476.2 ± 3.1
1941-BT	ND	13.5 ± 1.0	4.2 ± 0.2	5.9 ± 0.3
Δ*SSP_Lrp*-BT	ND	10.4 ± 0.9	8.4 ± 0.1	17.5 ± 0.1

*ND* not determined

The truncated SSP_Lrp was determined whether it was beneficial to increase BT (ISPs, main components) yield. The *ist* gene was integrated into the genome of Δ*SSP_Lrp*-SP and *S. spiramyceticus* 1941 to obtain the BT producing strains, Δ*SSP_Lrp*-BT and 1941-BT. Fermentation products of the Δ*SSP_Lrp*-BT and 1941-BT strains were analyzed by HPLC (Fig. 5b and Table 2). The concentration of ISP I was too low to detect its peak in HPLC profiles. The yield of ISP II and ISP III in Δ*SSP_Lrp*-BT was greater than that in the 1941-BT strain (17.5 ± 0.1 μg/L versus 5.9 ± 0.3 μg/L, respectively). The proportion of ISP III in the 1941-BT strain was 4.2% greatly increased to 8.4% in Δ*SSP_Lrp*-BT. The results showed that deletion of the l-Leu putative binding domain in the SSP_Lrp protein was favorable to the yields of SP or BT, particularly the proportions of SP III or ISP III. The 3-O-acyltransferase of SP biosynthesis can recognize both acetyl-CoA and propionyl-CoA substrates to produce SP II and SPIII, respectively. So, more propionyl substrates were produced in the truncated SSP_Lrp mutant, which are probably from amino acids catabolism.

### Involvement of the SSP_Lrp in BCAAs catabolism in *S. spiramyceticus* 1941

**Fig. 6:**
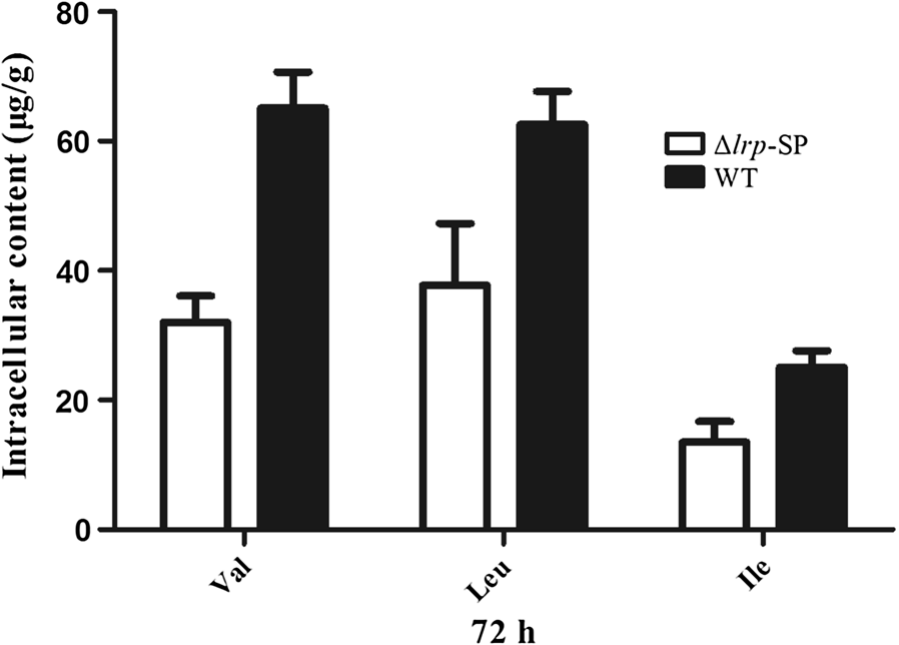
Histogram of the detection of intracellular BCAAs in Δ*SSP_Lrp*-SP mutant and *S. spiramyceticus* 1941 strains. WT: *S. spiramyceticus* 1941 (n = 2)

The intracellular and extracellular contents of amino acids in the Δ*SSP_Lrp*-SP mutant were detected. The results showed that intracellular BCAAs concentration dramatically decreased in the Δ*SSP_Lrp*-SP strain, compared with the *S. spiramyceticus* 1941 (Fig. 6). Specifically, the intracellular contents of Valine (Val), leucine (Leu), and isoleucine (Ile) decreased from 65.1 ± 5.6, 62.6 ± 5.1, and 25.1 ± 2.5 μg/g to 32.0 ± 4.1, 37.8 ± 9.5, and 13.5 ± 3.2 μg/g, respectively (Additional file 1: Table S3). The other diminished amino acids in cell include α-Alanine (α-Ala), glycine (Gly) and Threonine (Thr). However, the extracellular contents of the above 6 amino acids in the Δ*SSP_Lrp*-SP strain were similar to those in *S. spiramyceticu*s 1941 by analysis of fermentation broth supernatants (Additional file 1: Table S4). The increased catabolism of BCAAs could provide more substrates, such as acyl-CoA, propionyl-CoA and isovaleryl-CoA, to post-PKS modification of SP or BT biosynthesis; and the catabolic pathway Val and Ile were inclined to synthesis of propionyl-CoA which was the substrate for 3-O-acyltransferase to produce SP III.

### Global regulatory role of SSP_Lrp in *S. spiramyceticus* 1941

Comparative analysis of transcriptome data from RNA-seq of the Δ*SSP_Lrp*-SP and *S. spiramyceticus* 1941 strains found 45 genes with obvious differences in expression, including 12 significantly up-regulated and down-regulated genes (Table 3). The genes associated with macrolide antibiotic biosynthesis (comp4553), acyl-CoA metabolic process (com8771), and aspartic acid metabolism (comp9112), were distinctly detected in Δ*SSP_Lrp-*SP mutant, but not in the *S. spiramyceticus* 1941. The comp4553 sequence was found to locate in type I polyketide synthase (PKS) gene of the SP biosynthetic gene cluster. The genes related to comp8771 and comp9112 were inferred to involve in biosynthesis of the carboxylic acid units as the substrates of PKS. The expression of genes encode TPP-dependent pyruvate dehydrogenase (comp4959), Sigma24 family factor (comp5288) and a hydrolase involved in isoprenoid biosynthesis (comp1450) were improved 4.6, 3.6, and 2.5 folds in Δ*SSP_Lrp-*SP strain than that in the *S. spiramyceticus* 1941. On the contrary, the transcription of genes associated with ribosomal S7 and S8 biosynthesis, terpenoid biosynthesis, glutamate synthase and glutamine synthetase, were hardly detected in the Δ*SSP_Lrp*-SP mutant at fermentation 72 h. The other down-regulated gene was an AcrR family transcriptional regulator.

**Table 3:** Differentially expressed genes between the Δ*SSP_Lrp*-SP strain and *S. spiramyceticus* 1941

ID	*P* value	GO term	Direction of change	Fold change
comp4553	0.005	Acyl transferase; macrolide antibiotics biosynthetic pathway	Up	Infinity
comp8771	0.025	Acyl-CoA thioesterase; acyl-CoA metabolic process	Up	Infinity
comp9112	0.029	Aspartic acid 1- decarboxylase	Up	Infinity
comp4959	0.028	TPP-dependent pyruvate dehydrogenase	Up	4.6
comp5288	0.005	Sigma24 family factor	Up	3.6
comp1450	0.044	Hydrolase; isoprenoid biosynthesis pathway	Up	2.5
comp1000	0.045	Ribosomal protein S8	Down	Infinity
comp7405	0.023	Ribosomal protein S7	Down	Infinity
comp10453	0.029	Cytochrome P450, sesquiterpenoid and triterpenoid biosynthesis	Down	Infinity
comp4083	0.023	Glutamine synthetase	Down	Infinity
comp9736	0.043	Glutamate synthase	Down	Infinity
comp1697	0.046	DNA-binding transcriptional regulator, AcrR family	Down	5.1

**Fig. 7:**
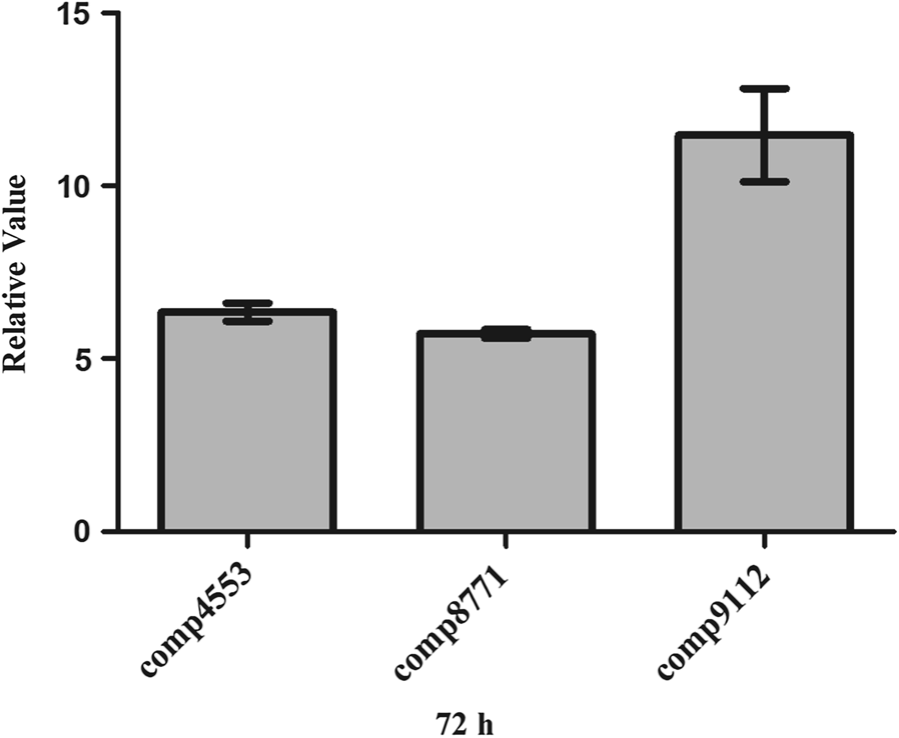
qPCR of genes in the Δ*SSP_Lrp*-SP and *S. spiramyceticus* 1941 strains. WT: *S. spiramyceticus* 1941 (n = 3), 4353: comp4553, 87711: comp8771, 9112: comp9112

The expression of comp4553, and comp8771 and comp9112 genes was further validated repeatedly by qPCR (Fig. 7). The results showed that the expression levels of these three upregulated genes in the Δ*SSP_Lrp*-SP mutant were more than five times higher than those in the wild-type strain (comp4553: 6.34-fold increase; comp8771: 5.72-fold increase; comp9112: 11.47-fold increase).

## Discussion

SSP_Lrp gene, outside the SP biosynthetic gene cluster, was identified from the genomic DNA sequence of *S. spiramyceticus* 1941, as a global regulator involved in the regulation of BT or SP biosynthesis. In this study, we showed that SSP_Lrp protein specifically bound to the promoter regions of *acyB2*, *bsm23*, and *bsm42* genes which are positive regulatory genes located in BT biosynthetic gene cluster. Therefore, we assumed that the SSP_Lrp regulator was a higher hierarchy member in regulatory networks of SP and BT biosynthesis. But the SP production of SSP_Lrp-null mutant (Δ*SSP_Lrp*) was just improved a little and with similar phenotype to wild type. However, high expression of SSP_Lrp in *S. spiramyceticus* 1941 significantly decreased the yield of SP. By deleting the l-Leu putative binding domain of SSP_Lrp in *S. spiramyceticus* 1941, the production of SP and BT were evidently increased, furthermore, significantly improved on the SP III and ISP III components. These results demonstrated that SSP_Lrp played a negative role involved in SP or BT biosynthesis. It may depress the transcription of positive regulatory genes or lessen the precursor supply of SP biosynthesis.

Lrp is a global transcription regulator that affects expression of a number of genes, acting as both an activator and a repressor with diverse co-effector binding in different bacteria. Akasaka et al. [20] reported complete deletion of the *lrp* gene in *G. europaeus*, and BCAAs were found to be increased in the mutant cells compared with the original strain; however, growth retardation was also observed. In contrast, when the C-terminal ligand binding domain of Lrp was deleted, the growth rate of the strain was normal, however, BCAAs content was still increased. However, deletion of the *lrp* gene in *S. erythraea* resulted in decreased BCAAs content compared with the original strain, but normal cell growth [20]. The *lrp* null mutant of *Komagataeibacter europaeus* showed significantly inhibited expression of the gene encoding S-adenosylmethionine synthetase and extensively reduced spermidine efflux; however, both of these parameters were significantly upregulated in the Kelrp mutant lacking the putative C-terminal ligand-sensing domain [16]. Δ*SSP_Lrp*-SP mutant lacking the putative C-terminal ligand binding domain tended to improve the yield of SP, especially the III components, with the normal cell growth and sporulation.

Lrp was a global regulator of metabolism associated with the feast-or-famine response in *E. coli* [17]. The SSP_Lrp in *S. spiramyceticus* 1941 is assumed to have a similar role. The truncated SSP_Lrp may break the balance of amino acids synthesis and catabolism, which possibly direct metabolic flux of amino acids into secondary metabolites, including the SP or BT biosynthesis. The SSP_Lrp was verified to involve in the catabolism of BCAAs in *S. spiramyceticus* 1941. In the Δ*SSP_Lrp*-SP stain, the intracellular concentration of BCAAs decreased significantly compared with that in *S. spiramyceticus* 1941. The catabolic pathway of BCAAs could provide the more substrates for SP or BT biosynthesis. The yields of SP III and ISP III increased prominently in Δ*SSP_Lrp*-SP and Δ*SSP_Lrp*-BT strains probably due to providing more propionyl-CoA from catabolic pathway of BCAAs, such as Ile and Val, for propanylation at C3 position to form SP III or ISP III. In addition, the Aspartic acid 1-decarboxylase gene (comp9112) is related to the beta-alanine synthesis, and catabolic pathway of beta-alanine is also associated with –COCH_2_CH_3_ formation in a series of enzymatic reactions in bacteria.

Transcriptome analysis and qPCR results helped us find out that comp4553, encoding KS domain of PKS, has been initiated at 72 h in the fermentation of the Δ*SSP_Lrp*-SP stain, but it was detected little in *S. spiramyceticus*. This result indicated the SP biosynthesis started earlier in the Δ*SSP_Lrp*-SP stain than wild type strain. In addition, enhancing the genes related to isoprenoid biosynthesis, pyruvate dehydrogenase, Sigma24 family factor, could affect the metabolic flux of bacteria in vivo, providing more energy for secondary metabolism. On the other hand, down-regulation of ribosomal protein, primary nitrogen metabolism and some terpenoids biosynthesis could save the biosynthetic substrates and energy to the secondary metabolism, potentially directing the metabolic flux into the SP or BT biosynthesis. Glutamate synthase and glutamine synthetase functioned in nitrogen metabolism are involved in primary metabolism. Glutamine synthetase participates in amino acid metabolism and functions as a metabolic switch during nitrogen assimilation [30]. The AcrAB multidrug efflux pump is negative controlled AcrR regulator [31]. Down-regulation of AcrR would produce more AcrAB to recognize and extrude a wide range of antibiotics, and improve the intrinsic antibiotic resistance in *S. spiramyceticus*. Taken together, all these mechanisms endorse SP and BT production.

## Conclusions

In this study, the SSP_Lrp protein encoded by *orf2* was identified from *S. spiramyceticus* and found to bind to the promoters of the three regulatory genes, *acyB2*, *bsm42*, and *bsm23*, which were positive regulators involved in SP and BT biosynthesis. When the co-effector binding domain of SSP_Lrp was deleted in SP- and BT-producing strains, the total production of SP and BT was apparently improved. Moreover, the BCAAs concentrations in truncated Lrp mutants were lower than those in the wild-type strain, although the concentrations of BCAAs were similar in the fermentation broths from the SSP_Lrp mutant and wild-type strains. Transcriptome and qPCR analyzes showed that genes related to SP and BT biosynthesis, including isoprenoid biosynthesis, macrolide antibiotic biosynthesis, alanine biosynthesis, and fatty acyl coenzyme A metabolism, showed higher expression in the Δ*SSP_Lrp*-SP and Δ*SSP_Lrp*-BT mutants compared with those in the corresponding wild-type strains. Interestingly, ISP III was greatly increased in the Δ*SSP_Lrp*-BT mutant, which could make it easier to meet the BT standard of quality criteria (BT end product to contain more than 30% ISP III). Furthermore, the deletion of this binding domain in Lrp executed in the existing BT high-yield strain would produce a new improved one for industrial applications. The stability and robustness of Δ*SSP_Lrp*-SP and Δ*SSP_Lrp*-BT strains should be evaluated in more detail in subsequent studies.

## Materials and methods

### Strains, plasmids, and growth conditions

All strains and plasmids used in this study are listed in Additional file 1: Table S1. Standard medium and culture conditions were used [32, 33]. The slant, seed culture, fermentation and bioassay medium for *S. spiramyceticus *1941 and its derivatives were described before [34]. Streptomyces strains were cultivated at 28 °C in soluble fermentation medium for isolation of total RNA and detection of concentration of amino acids. Soluble fermentation medium (per 100 mL) contained: dextrin, 5.0 g; NaCl, 1 g; MgSO_4_, 0.55 g; CaCO_3_, 0.5 g; NH_4_NO_3_, 0.7 g, KH_2_PO_4_, 0.065 g; CoCl_2_, 510^−5 ^g; with pH adjusted to 7.2 before autoclaving.

### Primers

All primers used in this study are listed in Additional file 1: Table S2.

### Heterologous expression and purification of SSP_Lrp

For heterologous expression of SSP_Lrp protein in *E. coli*, the SSP_Lrp gene was amplified by polymerase chain reaction (PCR) from the genome of *S. spiramyceticus* 1941 using the primer pair lrp-F/R and cloned into the pQE9 vector, generating an N-terminal His-tag fusion. The constructed plasmid pQE9-*SSP_Lrp* was introduced into *E. coli* BL21 (DE3), and protein expression was induced with isopropyl β-d-1-thiogalactopyranoside at a final concentration of 0.5 mM at 16 °C for 8–10 h. His6-tagged Lrp protein was extracted and purified on a Ni^2+^-NTA spin column (Qiagen, Valencia, CA, USA). The quality of the purified protein was estimated by sodium dodecyl sulfate polyacrylamide gel electrophoresis (SDS-PAGE). The protein concentration was determined using Bradford assays.

### Electrophoretic mobility shift assays (EMSAs)

EMSAs were performed as described previously [35]. Briefly, the intergenic segment between *ist* and *acyB2* and the promoter regions of *bsm23* (an *srm22* homolog) and *bsm42* (an *srm40* homolog) were amplified by PCR with their respective primers. For the binding reactions, the DNA probe at 0.4 ng was allowed to interact with different concentrations of Lrp in a mixture of 20 µL containing 10 mM Tris–Cl (pH 7.5), 50 mM NaCl, 1 mM ethylene diamine tetraacetic acid (EDTA), 4 mM dithiothreitol, 5% (v/v) glycerol, 0.1 ng/µL negative-control DNA, and 300 µg/mL acetylated bovine serum albumin. After incubation for 20 min at room temperature, 10 µL of each reaction was loaded onto a 5%polyacrylamide gel in Tris–borate-EDTA buffer (pH 8.7). The gels were stained in GelRed solution (BioTium) for 20 min, washed twice with deionized H_2_O, and scanned with a gel imaging system (Tanon).

### Gene deletion, complementation, and overexpression

Gene truncation in *S. spiramyceticus* 1941 was performed using the CRISPR-cas9 system [36]. With *S. spiramyceticus* 1941 genomic DNA as a template, 1.3-and 1.0-kb DNA fragments flanking the SSP_Lrp gene were amplified by PCR using the primer pairs *lrp*-AF/*lrp*-109LysR and *lrp*-109LysF/*lrp*-AR. The two PCR products were ligated by overlapping PCR, then digested with *Xba*I and *Hind*III, and then ligated into the corresponding sites of pKCcas9dO, yielding pKC-*SSP_Lrp*. By homologous chromosomic recombination with linearized fragments, a 120-nt fragment of the SSP_Lrp gene was deleted from the corresponding code of Lys109 in *S. spiramyceticus* 1941, and the truncated Lrp lacked the deduced l-Leu binding domain. The desired mutant, named Δ*SSP_Lrp-*SP, was further confirmed by PCR analysis using the primers *lrp*-CF/R and DNA sequencing.

SSP_Lrp gene was inactivated by gene replacement through the insertion of the apramycin resistance cassette *aac(3)IV* constructed in *Escherichia coli*/*Streptomyces* shuttle vector *p*GH112 with the unstable SCP2* replicon. The homologous arms of SSP_Lrp gene were obtained by primer pairs of *lrp*-LF/LR and *lrp*-RF/RR. The apramycin resistance cassette was excised from *p*UC-Am by restriction digestion with *Bam*HI and *Pst*I. The two arms fragments and apramycin resistance cassette were sub-cloned into the *p*GH112 vector to obtain resulting plasmid *p*GH–*SSP_Lrp*. After restriction digestion analysis and PCR confirmation, pGH–*SSP_Lrp* plasmid was introduced into *S. spiramyceticus* 1941 by protoplast transformation on R2YE plates. Transformants with apramycin resistance were streaked on plates with apramycin resistance continuously for five rounds to generate single-crossover mutants. Using double-crossover homologous recombination, the partial *orf* sequence of SSP_Lrp was replaced by the apramycin resistance cassette. Double-crossover transformants were thiostrepton-sensitive (Tsr^S^) and apramycin-resistant (Am^R^). The resulting Δ*SSP_Lrp* mutants were then verified by PCR and DNA sequencing.

For overexpression of SSP_Lrp in the ∆*SSP_Lrp-*SP mutant and wild-type strains, the constitutive ermE*p promoter was used to drive expression of SSP_Lrp gene. The 614-bp fragment containing promoterless SSP_Lrp gene was amplified from *S. spiramyceticus* 1941 genomic DNA using the primer pair *lrp-*EF and *lrp*-ER. The ermE*p promoter was excised from *p*UC-ermE*p by *Eco*RI and *Bam*HI, and the promoterless SSP_Lrp gene fragment was digested with *Bam*HI and *Xba*I. Both the ermE*p promoter and the SSP_Lrp gene coding fragment were ligated together with *Eco*RI- and *Xba*I double digested *p*SET152. The resulting *p*SET- ermEp*-*SSP_Lrp* was introduced into the ∆*SSP_Lrp*-SP mutant and wild-type strains to generate Δ*SSP_Lrp*-SP–C and 1941-C strains, respectively.

### Fermentation and detection of SP

For SP and BT production, the spores of *S. spiramyceticus *1941 and its derivatives were inoculated in seed culture (50 mL in a 250 mL flask) and incubated at 28 °C in an orbital shaker at 220 r.p.m for 2–3 days. Then, 2 mL of seed culture was transferred to fermentation medium (50 mL in a 500 mL flask) and incubated at 28 °C and 220 rpm for 4 days. All fermentation cultures were grown at 220 rpm and 28 °C for 6 days. SP and BT was extracted from the fermentation cultures and analyzed using a Waters 1500-series HPLC and Waters ACQuity UPLC (Agilent extend-C18 column, 5 µm, 250 × 4.6 mm), as previously described [34].

### Transcriptome analysis and quantitative real-time PCR

This transcriptome analysis was performed by OE Biotech (Shanghai, People’s Republic China). *P* value < 0.05 and fold Change > 2 or fold Change < 0.5 set as the threshold for significantly differential expression. Genes with more than twofold change and *P* value < 0.05 were defined as significantly regulated genes.

Total RNAs were isolated from *Streptomyces spiramyceticus* 1941 and Δ*SSP_Lrp-*SP strain after growth for 72 h in soluble fermentation medium using an RNA extraction/purification kit (Tiangen), and the RNA concentration was determined using a microplate reader (DeNoVix). Isolated RNA (400 ng) was treated with DNase I (Novagen), and reverse transcription was performed using a cDNA synthesis kit (Genstar). Quantitative real-time PCR was performed on a Light Cycler 96 (Roche) with FastStart Essential DNA Green Master (Roche). All qPCR gene‐specific primers were designed to produce ∼150 bp long amplicons and all reactions were performed in triplicate for three different samples using gene specific primers (Additional file 1: Table S2). The 16S RNA gene from *S. spiramyceticus* 1941 was used as the internal control to normalize samples. PCR program: 96 °C 1 min (96 °C 30 s, 61 °C 30 s, 72 °C 1 min) 40 cycles, 72 °C 10 min. Melting-curve analysis was performed to check the specificity of PCR amplification. Melting-curve analysis was performed to check the specificity of PCR amplification. Cycle threshold (Ct) values were obtained from the exponential phase of PCR amplification and genes’ expression was normalized against the genes expression of 16S RNA to generate a ΔCt value (Ct of target gene–Ct of endogenous control). The change in the genes’ expression was calculated using 2^−ΔΔCt^ method.

### Measurement of intracellular and extracellular amino acid concentrations

*S. spiramyceticus* 1941 and Δ*SSP_Lrp-*SP were grown in soluble fermentation medium, and havested at 72 h. A HITACHI l-8900 amino acid analyzer was used for quantification of intracellular and extracellular amino acids from in with the ninhydrine colorimetric method [21, 37].

### Statistical analysis

All data in this study are presented as means ± standard errors of the means (SEMs) and analyzed by Student’s t-tests. Differences with *P* values of less than 0.05 were considered significant.

## Additional file

**Additional file 1: Table S1.** Strains and plasmids used in this study.** Table S2.** Primers used in this study.** Table S3.** Intracellular amino acids of the ΔSSP_Lrp-SP and S. spiramyceticus 1941 strains.** Table S4.** The extracellular amino acids of ΔSSP_Lrp-SP strain and S. spiramyceticus 1941.
