# Supplementary material for: Engineering of leucine-responsive regulatory protein improves spiramycin and bitespiramycin biosynthesis
Source: Microb Cell Fact. 2019 Feb 19;18:38. doi: 10.1186/s12934-019-1086-0 (PMC6379999; doi:10.1186/s12934-019-1086-0)
Supplement: Supplementary file 1 — Additional file 1: Table S1. Strains and plasmids used in this study. Table S2. Primers used in this study. Table S3. Intracellular amino acids of the ΔSSP_Lrp-SP and S. spiramyceticus 1941 strains. Table S4. The extracellular amino acids of ΔSSP_Lrp-SP strain and S. spiramyceticus 1941. [file 12934_2019_1086_MOESM1_ESM.docx]

**Supplementary material**

**1. Table S1 Strains and plasmids used in this study**

| **Strains/plasmid** | **Genotype and characters** | **Sources** |
| --- | --- | --- |
| **Strains** |  |  |
| 1941 | Wild type *S. spiramyceticus* 1941 | China Pharmaceutical  Culture Collection， CGMCC 8279 |
| 1941-C | 1941 containing pSET-*ermEp**-*SSP_Lrp* plasmid | This study |
| 1941-BT | *S. spiramyceticus* 1941 containing *p*SET-*ermEp**-*ist* plasmid | This study |
| Δ*SSP_Lrp-*SP | Truncated SSP_Lrp gene in 1941 | This study |
| Δ*SSP_Lrp*-SP-C | Δ*SSP_Lrp*-SP containing pSET-*ermEp**-*lrp* plasmid | This study |
| Δ*SSP_Lrp-*BT | Δ*SSP_Lrp-*SP containing *p*SET-*ermEp**-*ist* plasmid |  |
| Δ*SSP_Lrp* | Deletion of SSP_Lrp gene in 1941 | This study |
| DH5α | F recA lacZM15 | Invitrogen |
| *E. coli* JM109 | *rec*A *sup*E44 *end*A1 *hsd*R17 *gyr*A96 *rel*A1 *thi* Δ(*lac*-*pro*AB) F’[*tra*D36 *pro*AB+ *lac*Iq *lac*ZΔM15] | TransGen Biotech in Beijing |
| *E. coli* ET12567/*p*UZ8002 | *dam*^-^ *dcm*^-^ *hsd*M^-^, *p*UZ8002 | Kieser et al., 2000 |
| **Plasmids** |  |  |
| *p*SET152 | *E. coli-Streptomyces*shuttle vector，Am^R^ | Kieser et al., 2000 |
| *p*UC-*ermE*p* | pUC19 plasmid carrying *ermE*p* promoter | Store in the Lab |
| *p*UC-Am | pUC19 plasmid carrying the apramycin resistant gene *aac(3)IV* | Store in the Lab |
| *p*SET-*ermEp**-*ist* | *p*WHM3 carrying *ist* gene inserted downstream of *ermEp** promoter | This study |
| *p*SET- *ermEp**-*SSP_Lrp* | *p*SET152 carrying SSP_Lrp gene inserted downstream of *ermEp** promoter | This study |
| *p*KCcas9dO | *acc(3)IV*, pSG5, *tipA*-*Scocas9*, j23119, *actII-orf4* guide-RNA, homologous region flanking *act-orf4* | [Huang, et al., 2015 ] |
| *p*KC-*SSP_Lrp* | pKCcas9dO *carrying the* truncated SSP_Lrp gene | This study |
| *p*GH-*SSP_Lrp* | Suicide plasmid for SSP_Lrp gene disruption | This study |
| *p*QE9 | AmpR，His-Tag | addgene/novagen |
| *p*QE9-*SSP_Lrp* | pQE9 derivative for expression of SSP_Lrp gene | This study |

**Table S2 Primers used in this study**

| **Oligos** | Sequence (5’-3’) | Use |
| --- | --- | --- |
| *lrp*-F | CGGGATCCACCGGATATTCACCGGACGCC | Expression of SSP_Lrp gene in *E. coli* |
| *lrp*-R | CCCAAGCTTGCGGCTGAGCGCCCTGCGCG |  |
| *lrp*-sgRNA-F | GGACTAGTCATGTCCGCGAGCGCCGTGAGTTTTAGAGCTAGAAAT | The cut site of SSP_Lrp gene by Cas9 |
| *lrp-*sgRNA-R | GCTCTAGACTCAAAAAAAGCACCGACTCGG |  |
| *lrp*-AF | GCTCTAGAAGCTGGCGCACGAGTTCTT | Construction of pKC-*SSP_Lrp* plasmid for deletion of the co-factor binding domain |
| *lrp*-AR | CCCAAGCTTGTCGCTGGAGGCGGAGTT |  |
| *lrp*-109LysR | GCACGGCTCCGCGGTGCGGCACCACATGACGGGGATCACAGCACGAAGCA |  |
| *lrp*-109LysF | TCCCCGTCATGTGGTGCCGCACCGCGGAGCCGTGC |  |
| *lrp*-LF | CCGGAATTCGTACGCGGAGCGGCTGAT | Construction of pGH-*SSP_Lrp* plasmid for deletion of the SSP_Lrp gene |
| *lrp*-LR | AAAACTGCAGTGAATATCCGGTCATGCG |  |
| *lrp*-RF | CGCGGATCCGTCGCGGCCCGCTCGATGAA |  |
| *lrp*-RR | CTAGTCTAGATCGCTGGAGGCGGAGTTGGT |  |
| *lrp*-CF | GTGGGGGCCTGGGGAGTG | Confirmation of Δ*SSP_Lrp*-SP mutant by PCR |
| *lrp*-CR | ACCGCAGCGTGGGCATCG |  |
| *lrp-DF* | GCACTGGCGACGGTACGAGA | Confirmation of Δ*SSP_Lrp* mutant by PCR |
| *lrp-DR* | GCGACTGGATGCGTGATGTG |  |
| *lrp-*EF | CGCGGATCCGATTCCCCGGCTCTTCTCT | overexpression of SSP_Lrp *gene* |
| *lrp-*ER | CTAGTCTAGAACCGCAGCGTGGGCATCGTG |  |
| *lrp*-NF | AGGCTCCGCCCCCCTGACGA | EMSA analysis of nonspecific control DNA |
| *lrp*-NR | GAGAAAGCGCCACGCTTCCCG |  |
| *pIA*-F | GGGTGACCTCGCGGCGGACG | EMSA analysis of *ist* and *acyB2* intergenic sequence |
| *pIA*-R | TATGACCCTCACAAGCCGCT |  |
| *bsm23*-F | AGTCGCCGGTAGCTTATCC | EMSA analysis of *bsm23* promoter |
| *bsm23*-R | TACGAATACAATGACAGGTTA |  |
| *bsm42*-F | GGCAGGTAGGCCATTTGGG | EMSA analysis of *bsm42* promoter |
| *bsm42*-R | TCGTCATTCCTGCGAGTGG |  |
| *ist*-F | TCGGTGTGGGACGGACTGCT | Confirmation of Δ*SSP_Lrp*-SP and Δ*SSP_Lrp*-BT strain by PCR |
| *ist*-R | CGCGCCGGGGTCGTAGTGGT |  |
| 4553-F | GGGCGTGTCGGGGTCGGGCG | qRT-PCR analysis of comp4553 |
| 4553-R | CGGACGAACTGTGGGACCTG |  |
| 9112-F | CCGAGAGGCTGCCGCCTCAG | qRT-PCR analysis of comp9112 |
| 9112-R | GACCTGGTGATCCTCATCA |  |
| 8771-F | GCGCAGCCGGTCGACGTACC | qRT-PCR analysis of comp8771 |
| 8771-R | TCCACCAGCCGGAGGAGGCC |  |
| 16S-RNA-F | GGTAGAGCTTGTTGACGCAGAG | qRT-PCR analysis of 16S RNA as control |
| 16S-RNA-R | ATGAGGGCGAGGACAGCGATGC |  |

**Table S3.** Intracellular amino acids of the Δ*SSP_Lrp*-SP and *S. spiramyceticus* 1941 strains

| Name | Δ*SSP_Lrp*-SP (*μ*g/g) | 1941(*μ*g/g) |
| --- | --- | --- |
| α-Ala | 291.6±20.3 | 349.7±33.8 |
| Val | 32.0±4.1 | 65.1±5.6 |
| Leu | 37.8±9.5 | 62.6±5.1 |
| Ile | 13.5±3.2 | 25.1±2.5 |
| Pro | 8.8±3.2 | 7.8±0.4 |
| Gly | 9.5±3.8 | 35.9±5.2 |
| Ser | 14.1±2.6 | 17.7±0.1 |
| Thr | 11.0±2.9 | 24.2±0.1 |
| β-Ala | Not detectable | Not detectable |
| Met | 12.3±0.4 | 10.7±0.9 |
| Pyr | 28.8±2.5 | 25.9±2.5 |
| GABA | 42.8±2.3 | 43.0±6.8 |
| Cys | Not detectable | Not detectable |
| Glu | 1710.2±118.1 | 1671.4±578.8 |
| Phe | 48.1±2.1 | 55.2±1.1 |
| Asn | 50.0±0.2 | 50.20±0.1 |
| Orn | 20.2±0.7 | 19.5±0.8 |
| Lys | 116.4±4.0 | 116.9±2.4 |
| Tyr | 75.8±0.2 | 75.8±0.2 |
| Trp | 34.7±5.1 | 29.6±0.3 |

**Table S4** The extracellular amino acids of Δ*SSP_Lrp*-SP strain and *S. spiramyceticus* 1941

| Name | Δ*SSP_Lrp*-SP (μg/ ml) | *S. spiramyceticus* 1941  (μg/ ml) |
| --- | --- | --- |
| α-Ala | 0.19±0.07 | 0.05±0.01 |
| Val | 0.16±0.05 | 0.08±0.01 |
| Leu | 0.48±0.04 | 0.41±0.01 |
| Ile | 0.2±0.05 | 0.14±0.01 |
| Pro | 0.13±0.03 | 0.06±0 |
| Gly | below | below |
| Ser | 0.91±0.02 | 0.88±0 |
| Thr | 0.28±0.05 | 0.24±0 |
| β-Ala | 0.41±0 | 0.41±0 |
| Met | 4.64±0.07 | 4.64±0 |
| Pyr | 2.45±0.04 | 2.41±0 |
| GABA | 2.82±0.04 | 2.79±0.01 |
| Cys | below | below |
| Glu | 5.73±0 | 5.73±0 |
| Phe | 9.94±0.01 | 9.94±0.01 |
| Asn | below | below |
| Orn | 3.99±0.01 | 3.99±0 |
| Lys | below | below |
| Tyr | below | below |
| Trp | below | below |
